# Assessment of ultrasonographic features of polycystic ovaries is associated with modest levels of inter-observer agreement

**DOI:** 10.1186/1757-2215-2-6

**Published:** 2009-06-10

**Authors:** Marla E Lujan, Donna R Chizen, Andrew K Peppin, Anita Dhir, Roger A Pierson

**Affiliations:** 1 Division of Nutritional Sciences, Cornell University, Ithaca, USA; 2 Division of Obstetrics, Gynecology & Reproductive Sciences, University of Saskatchewan, Saskatoon, Canada; 3 Division of Radiology & Diagnostic Imaging, University of Alberta, Edmonton, Canada; 4 Division of Academic Department of Medical Imaging, University of Saskatchewan, Saskatoon, Canada

## Abstract

**Background:**

There is growing acceptance that polycystic ovaries are an important marker of polycystic ovary syndrome (PCOS) despite significant variability when making the ultrasound diagnosis. To better understand the nature of this variability, we proposed to evaluate the level of inter-observer agreement when identifying and quantifying individual ultrasonographic features of polycystic ovaries.

**Methods:**

Digital recordings of transvaginal ultrasound scans performed in thirty women with PCOS were assessed by four observers with training in Radiology or Reproductive Endocrinology. Observers evaluated the scans for: 1) number of follicles ≥ 2 mm per ovary, 2) largest follicle diameter, 3) ovarian volume, 4) follicle distribution pattern and 5) presence of a corpus luteum (CL). Lin's concordance correlation coefficients and kappa statistics for multiple raters were used to assess inter-observer agreement.

**Results:**

Agreement between observers ranged from 0.08 to 0.63 for follicle counts, 0.27 to 0.88 for largest follicle diameter, 0.63 to 0.86 for ovarian volume, 0.51 to 0.76 for follicle distribution pattern and 0.76 to 0.90 for presence of a CL. Overall, reproductive endocrinologists demonstrated better agreement when evaluating ultrasonographic features of polycystic ovaries compared to radiologists (0.71 versus 0.53; p = 0.04).

**Conclusion:**

Inter-observer agreement for assessing ultrasonographic features of polycystic ovaries was moderate to poor. These findings support the need for standardized training modules to characterize polycystic ovarian morphology on ultrasonography.

## Background

Polycystic ovary syndrome (PCOS) is a common endocrine disorder of unknown cause [1]. Epidemiological studies have estimated a prevalence of 6.5 to 8% using biochemical and/or clinical evidence [1] while studies involving ultrasonographic evidence of polycystic ovaries have reported a prevalence of 20% or more [2]. PCOS is characteristically heterogeneous in its clinical presentation and therefore, much debate remains regarding consensus diagnostic criteria for the syndrome [3]. Historically, the combination of androgen excess and oligo-amenorrhea has been considered the hallmark of PCOS by North American standards [4]. By contrast, British and European standards have based the diagnosis primarily on ultrasonographic evidence of polycystic ovaries [5]. Clarifying diagnostic criteria for PCOS has significant implications for the early identification and intervention of this condition. Early diagnosis and intervention is warranted since there is considerable evidence that women with PCOS are at increased risk for infertility, dysfunctional uterine bleeding, metabolic syndrome, type II diabetes and cardiovascular disease [6,7]. There is also growing evidence for increased risk of obstructive sleep apnea, depression, nonalcoholic fatty liver disease and certain cancers [8-11].

In 2003, ultrasonographic evidence of polycystic ovaries was formally incorporated as a diagnostic marker of PCOS at a joint meeting of the European Society for Human Reproduction and Embryology (ESHRE) and the American Society for Reproductive Medicine (ASRM) [6,7]. Inclusion of an ovarian marker was based on substantial evidence that most women who presented with clinical and biochemical features of PCOS had polycystic ovaries on ultrasound [12-14]. The current ultrasound guidelines supported by ESHRE/ASRM consensus characterize the polycystic ovary as containing 12 or more follicles measuring 2 – 9 mm and/or an increased ovarian volume of >10 cm^3 ^[15]. Unlike the widely used criteria previously proposed by Adams and colleagues [16], a subjective assessment of stromal echogenicity and follicle distribution pattern is not included. The cutoff value for an increased ovarian volume was derived from cumulative reports of a larger mean volume for polycystic ovaries compared to a mean volume of <10 cm^3 ^for normal ovaries [17]. The cutoff of ≥12 follicles throughout the entire ovary, and not a single plane, was based on a report demonstrating this value to have 99% specificity and 75% sensitivity in distinguishing between polycystic and normal ovaries in women of reproductive age [15].

While there is growing agreement that polycystic ovaries represent an important component of the clinical presentation of PCOS, it is important to acknowledge that significant inter- and intra-observer variability has been reported when making the ultrasound diagnosis [18]. In an analysis of 54 ovarian scans in which images of 27 polycystic and normal ovaries were duplicated and randomized for post-hoc evaluation by four experienced observers, a diagnosis of polycystic ovarian morphology was agreed upon only 51% of the time while observers agreed with himself/herself only 69% of the time [18]. In their study, Amer et al. defined the polycystic ovary as having ≥10 follicles (2 – 8 mm) in a single plane, an ovarian volume ≥12 cm^3 ^and a bright echogenic stroma. The high degree of variability in making the diagnosis suggested that the ultrasound criteria employed were either too subjective or too insensitive to allow for good agreement among observers [17]. The extent to which any of the ultrasound criteria contributed to the subjectivity of the diagnosis was not assessed and to date, we are unaware of any other study that has attempted to further evaluate subjectivity in the ultrasound diagnosis of polycystic ovaries.

In the present study, we attempted to determine where discrepancies in the evaluation of polycystic ovaries might lie by determining the level of inter-observer agreement associated with the assessment of individual ultrasonographic aspects of polycystic ovarian morphology such as total follicle count, largest follicle diameter, ovarian volume, follicle distribution pattern and presence of a corpus luteum. Given past reports of significant variability in diagnosing polycystic ovaries, we hypothesized that agreement when evaluating ultrasonographic features of polycystic ovaries would be poor even among experienced medical imaging specialists with training in Radiology or Reproductive Endocrinology.

## Methods

### Study subjects

Thirty women diagnosed with PCOS using the 2003 international consensus guidelines [6,7] of having two of three characteristics: 1) oligo- or anovulation (menstrual cycles <21 or >38 days)[19], 2) clinical and/or biochemical evidence of hyperandrogenism (modified Ferriman-Gallwey score ≥ 8 [20] and/or a free androgen index ≥ 4 [21]), 3) polycystic ovaries on ultrasound (≥12 follicles measuring 2 – 9 mm in diameter or an ovarian volume >10 cm^3^)[17], were enrolled in the study. Subjects ranged in age from 18 to 35 and could not have used hormonal contraception, fertility medications or valproate in the three months prior to enrolment. Subjects were screened for the absence of hyperprolactinemia, hypercortisolemia, thyroid dysfunction and 21-hydroxylase deficiency. The ability to visualize at least one ovary by transvaginal ultrasonography was required for inclusion in the study.

### Transvaginal ultrasonography

A single transvaginal ultrasound scan was performed at a random time (during the menstrual cycles) in subjects reporting absent, irregular or regular periods. Scans were performed by a single ultrasonographer using an UltraSonix RP ultrasound scanner equipped with a 9-MHz transvaginal transducer (UltraSonix, Version 2.3.5, Vancouver, BC). Each ovary was visualized and anatomic orientation with respect to the utero-ovarian ligament was established. Ovaries were scanned from the inner to outer margins in both the transverse and sagittal planes. Real-time ultrasound scans were digitally recorded (i.e., audio-video interleaved file format) and files later transferred to a custom-designed database for post-hoc image analysis.

### Randomization of ultrasonographic image files

Digital video clips of thirty individual ovaries (one from each subject) were selected for analysis from the sixty ovaries scanned. All video clips selected for the inter-observer analysis were judged by two raters to have good or excellent resolution of the ovary prior to inclusion. Each ovarian case study was designated an electronic folder on the database and each folder contained two digital video clips of the ovary in question – one clip represented a sweep through the ovary in the transverse plane and the other represented a sweep through the ovary in the sagittal plane. Links to these thirty folders were randomly generated for each of the four observers such that no observer reviewed the folders in the same order.

### Evaluation of ultrasonographic image files

Two senior Radiology residents (PGY 4 and PGY 5) and two clinician/scientists with training in Reproductive Endocrinology (a clinical reproductive endocrinologist and a fellow with training in transvaginal ultrasonography) reviewed the folders at computer workstations for the following primary endpoints: 1) total follicle count, 2) largest follicle diameter, 3) ovarian volume, 4) follicle distribution pattern and 5) presence of a corpus luteum (CL). For the follicle count endpoint, observers were asked to count the total number of follicles ≥ 2 mm in the entire ovary using one of the two video clips provided (i.e. clearly labeled "for follicle counts"). Observers were instructed to use both video clips to select the follicle with the largest diameter and to designate follicle distribution pattern. For the follicle distribution pattern endpoint, observers were to judge whether follicles in the ovary were predominantly distributed in a "peripheral" pattern or whether follicles were distributed more heterogeneously ("even") throughout the stroma. In instances where they felt that neither category could best describe the distribution pattern, a designation of "other" could be assigned. Observers were asked to calculate ovarian volume using the equation for a prolate spheroid [22] from measurements of the largest and widest diameters of the ovaries in the transverse and sagittal planes. Lastly, observers were instructed to determine the presence or absence of a corpus luteum using both video clips. Two complementary software programs (FRAME^© ^and SYNERGYNE 2^©^, Saskatoon, SK, Canada) were used to analyze the digital recordings. Video clips could be viewed at any speed or in direction including, frame-by-frame analysis. Colour/contrast adjustments and linear measurements could also be made on any frame of the video clip.

### Ethical considerations

This study was approved by the University of Saskatchewan Biomedical Research Ethics Review Board. All study procedures conformed to the Canadian Tri-Council Guidelines for Human Research and International Good Clinical Practice Guidelines. Informed consent was obtained from all study subjects.

### Statistical analyses

Descriptive statistics (mean ± SEM) for clinical, hormonal and metabolic features of the study subjects were garnered from clinical and laboratory medical records obtained at the time of evaluation for PCOS. Mean (± SEM) measurements of follicle counts, maximum follicle diameter and ovarian volume were tabulated and compared among observers using Tukey-Kramer's multiple comparisons tests and paired t-tests. Lin's concordance correlation coefficients (ρ) were used to assess inter-observer agreement for continuous measures [23] and kappa statistics for multiple raters (κ) were used to assess inter-observer agreement for discrete measures [24]. P and κ values that approximated 1 denoted perfect agreement, while values that approximated 0 denoted agreement no better than that by chance. Guidelines for evaluating level of agreement among scores were: >0.80 good, 0.60 – 0.80 moderate/fair, <0.60 poor [25].

## Results

### Subject demographics

Clinical, hormonal and metabolic features of the study participants are presented in Table 1. The average age of the participants was 28.3 ± 0.9 years and their mean BMI and waist circumference was 29.6 ± 1.3 kg/m^2 ^and 93.7 ± 2.7 cm, respectively. Forty-three percent of study subjects were obese (>30 kg/m^2^), 17% were overweight (26 – 30 kg/m^2^) and 40% were lean (≤25 kg/m^2^). Thirty-three percent of subjects reported menstrual cycles every 21 – 38 days, 30% reported cycles every 39 – 90 days and 37% reported cycles >90 days apart. Eighty-seven percent of subjects had elevated scores for hirsutism and/or an increased free androgen index. Only 13% of participants showed no clinical or biochemical signs of androgen excess. One subject demonstrated a mild case of impaired fasting glycemia (6.1 mmol/L) whereas the remaining participants demonstrated normal fasting glucose levels. Thirty percent of subjects were however, subsequently designated as insulin resistant as judged by an increased homeostatic model assessment of insulin resistance value.

**Table 1:** Clinical, hormonal and metabolic features of PCOS study subjects

	Mean ± SEM	Range	Normal Values
Age (yr)	28.3 ± 0.9	19 – 35	-
BMI (kg/m2)	29.6 ± 1.3	19.4 – 45.0	20 – 25
Waist Circumference (cm)	93.7 ± 2.7	70.0 – 123.0	< 88
Menstrual Cycle Length (d)	91.3 ± 15.0	28 – 365	21 – 38
LH:FSH	2.4 ± 0.3	0.6 – 7.6	< 2
mFG Score	9.3 ± 1.0	0 – 24	<8
Testosterone (nmol/L)	2.3 ± 0.2	1.0 – 5.0	< 2.5
SHBG (nmol/L)	45.9 ± 3.9	13.0 – 95.3	18 – 114
Free Androgen Index	6.2 ± 0.7	1 – 19	< 5
DHEA-S (μmol/L)	4.8 ± 0.3	1.8 – 8.8	0.9 – 12.0
Fasting Glucose (mmol/L)	4.8 ± 0.1	4.2 – 6.1	< 6.1
Fasting Insulin (pmol/L)	78.3 ± 10.4	21.0 – 205.0	14.0 – 100.0
HOMA-IR	2.9 ± 0.4	0.7 – 8.1	< 3

Normative values for the local health region are provided. Body mass index (BMI), luteinizing hormone (LH), follicle stimulating hormone (FSH), modified Ferriman-Gallwey (mFG), sex hormone binding globulin (SHBG), dehydroepiandrosterone sulfate (DHEA-S), homeostatic model assessment of insulin resistance (HOMA-IR).

### Continuous measures

Mean (± SEM) measurements for total follicle count, largest follicle diameter and ovarian volume reported by the four observers are compared in Table 2. Overall, the average number of follicles counted by the four observer was 33.5 ± 1.7, the mean largest follicle diameter was 8.0 ± 0.6 mm and the mean ovarian volume was 10.1 ± 0.5 cm^3^. Follicle counts varied among the four observers (p < 0.0001) with Observer 3 making significantly lower counts compared to each of the other three observers (p < 0.001). Largest follicle diameter (p = 0.090) and ovarian volume measurements (p = 0.650) did not differ among observers. When measurements were stratified for radiologists and reproductive endocrinologists, radiologists made lower follicle counts (27.6 ± 1.8 vs. 39.4 ± 2.0, p < 0.0001) and larger measurements for maximum follicle diameter (8.6 ± 0.4 vs. 7.4 ± 0.4, p = 0.003) and ovarian volume (10.5 ± 0.5 vs. 9.6 ± 0.6, p = 0.018) compared to reproductive endocrinologists.

**Table 2:** Ultrasonographic measurements of polycystic ovaries made by four observers

	**Observers**			
	
	1	2	3	4
Follicle Count	33.8 ± 1.6^a^	36.9 ± 2.5^a, c^	18.3 ± 1.0^b^	44.9 ± 3.3^c^
Largest Follicle (mm)	7.3 ± 0.6^a^	8.0 ± 0.6^a^	9.3 ± 0.6^a^	7.4 ± 0.5^a^
Ovarian Volume (cm^3^)	9.7 ± 0.8^a^	10.3 ± 0.8^a^	10.7 ± 0.8^a^	9.4 ± 0.9^a^

Significant differences for within row comparisons are denoted by different letters (p < 0.05). Observers 1 and 4 represent measurements made by reproductive endocrinologists. Observers 2 and 3 represent measurements made by radiologists.

**Figure 1:**
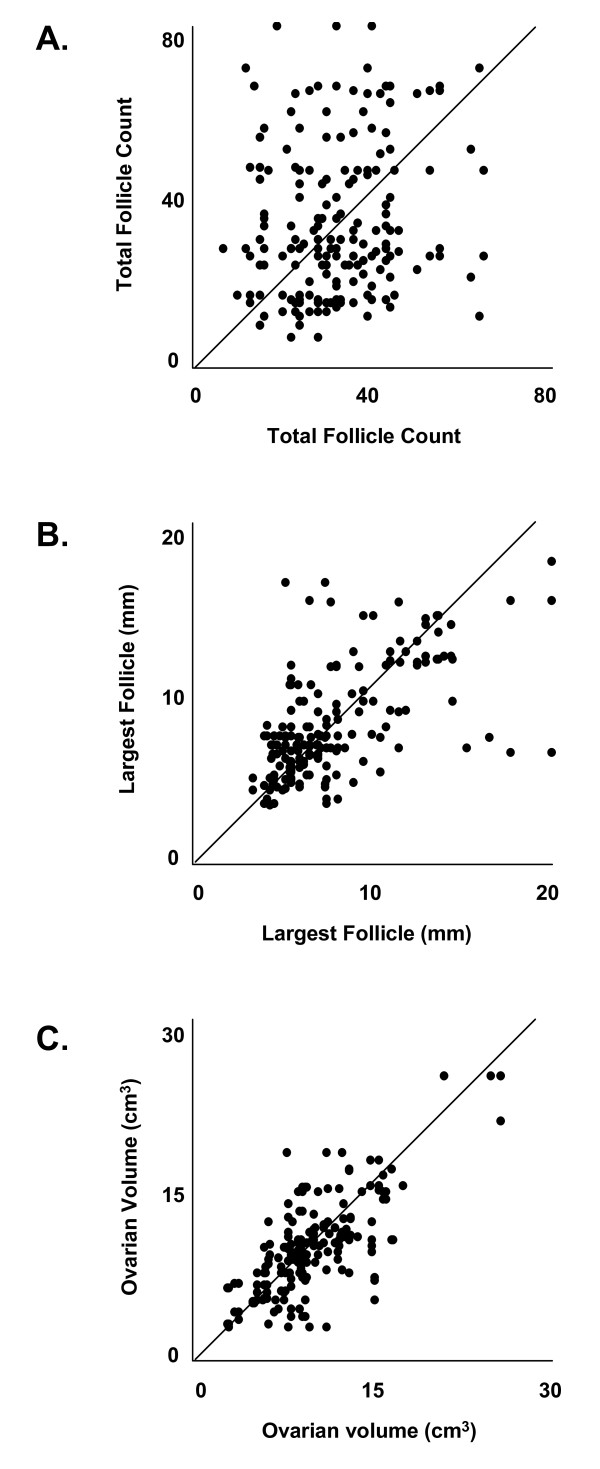
**Scatter plots of total follicle counts (A), largest follicle diameter measurements (B) and ovarian volume calculations (C) by all possible pair-wise combinations of the four observers are presented**. Perfect agreement between two observers corresponds to a slope of 1 (represented by the diagonal line). Inter-observer agreement was best for ovarian volume and poorest for total follicle counts.

Scatter plots of pair-wise agreement in follicle counts, largest follicle diameter measurements and ovarian volume calculations by four observers are presented in Figure 1. Perfect agreement between two observers corresponds to a slope of 1 (diagonal line). Inter-observer agreement was best for ovarian volume followed by largest follicle diameter and total follicle count, as judged by the predominance of points aggregating along the diagonal line. The corresponding levels of agreement among the observer pairs are summarized in Table 3. Agreement between observers ranged from 0.08 to 0.63 for follicle counts, 0.27 to 0.88 for largest follicle diameter and 0.63 to 0.86 for ovarian volume. Evaluators with training in Reproductive Endocrinology (represented by Observer Pair 1,4) demonstrated better agreement in follicle counts (0.27 vs. 0.16), largest follicle diameter (0.86 vs. 0.43) and ovarian volume (0.84 vs. 0.75) compared to those with training in general Radiology (represented by Observer Pair 2,3), respectively. In general, decreased levels of agreement were evident for the follicle count and largest follicle diameter endpoints when comparisons were made with Observer 3. Overall, inter-observer agreement was poor for continuous measures (overall ρ = 0.55)

**Table 3:** Level of pair-wise agreement among four observers assessing ultrasonographic features of polycystic ovaries

	**Concordance Correlation Coefficient**				**Kappa Statistic**		
		
Observer Pair	Follicle Count	Largest Follicle	Ovarian Volume	Average	Follicle Pattern	Corpus Luteum	Average
1,2	0.63	0.88	0.86	0.79	0.66	0.90	0.78
1,3	0.18	0.27	0.80	0.42	0.58	0.76	0.67
1,4	0.27	0.86	0.84	0.66	0.76	0.86	0.81
2,3	0.16	0.43	0.75	0.44	0.51	0.80	0.65
2,4	0.48	0.86	0.67	0.67	0.73	0.83	0.78
3,4	0.08	0.34	0.63	0.35	0.54	0.83	0.69

Average	0.30	0.61	0.76	0.55	0.63	0.83	0.73

Observer Pair 1,4 represent measurements made by reproductive endocrinologists, Observer Pair 2,3 represent measurements made by radiologists.

### Discrete measures

The level of agreement when assigning follicle distribution pattern and the presence of a CL is summarized in Table 3. Agreement between observers ranged from 0.51 to 0.76 for follicle distribution pattern and 0.76 to 0.90 for presence of a CL. Overall, inter-observer agreement was moderate for discrete measures (overall κ = 0.73). Evaluators with training in Reproductive Endocrinology (represented by Observer Pair 1,4) demonstrated better agreement when designating follicle distribution pattern (0.76 vs. 0.51) and presence of a CL (0.86 vs. 0.80) compared to those with training in general Radiology (represented by Observer Pair 2,3), respectively.

## Discussion

Our results showed that despite reproductive endocrinologists demonstrating better agreement than radiologists when evaluating ultrasonographic features of polycystic ovaries, overall inter-observer agreement for both groups was only moderate to poor. In the case of counting the total number of follicles throughout the entire ovary, agreement was alarmingly poor. This was in contrast to past reports of good agreement when multiple observers counted follicles using both real-time and stored transvaginal ultrasonographic imaging [26-28]. Good agreement in these studies was associated with counts that approximated 10 follicles per ovary [26,28]. In our current study, women diagnosed with PCOS by the ESHRE/ASRM criteria had counts that were generally in the order of 30 – 35 follicles. That we were counting more than three times as many follicles per ovary likely explains the lower levels of reliability reported by our group. The poor level of agreement for counting follicles may be interpreted to mean that follicle counts are too unreliable to be diagnostic. However, it is important to recognize that the current ultrasound guidelines only necessitate the ability to reliably count 12 follicles throughout the entire ovary [15]. Our data showed that observers were consistent in identifying at least 12 follicles per ovary; yet we were interested in assessing the reliability of total follicle counts since recent studies have suggested that a significantly higher threshold than 12 is needed to adequately discriminate between polycystic and normal ovaries [29]. Moreover, there is emerging evidence that ovarian morphology may reflect the degree of reproductive and metabolic disturbance in PCOS and therefore, give insight into the progression of the syndrome within an individual patient [30]. Future studies aimed at improving reliability in follicle counts will be needed to verify the validity and applicability of this ultrasonographic endpoint in the evaluation of PCOS.

In contrast to follicle counts, agreement when calculating ovarian volume was fair. This observation was consistent with several studies reporting good agreement when multiple observers assessed ovarian volume by ultrasonography [27,31-34]. Better agreement when calculating ovarian volume suggests that this endpoint may serve as a more reliable marker of polycystic ovaries than follicle counts. Unfortunately, there is significant debate regarding the sensitivity of increased ovarian volume as a diagnostic criterion for polycystic ovaries. The currently accepted cutoff of >10 cm^3 ^was associated with 98.2% specificity, but only 45% sensitivity, in discriminating between normal and polycystic ovaries [35]. Since 2003, both a lower threshold of 7 cm^3 ^[35] and a higher threshold 13 cm^3 ^[29] have been proposed as being more appropriate thresholds for polycystic ovarian morphology. Some of the controversy over a reliable diagnostic cut-off likely relates to inconsistent methods for determining ovarian volume. There is currently no consensus on the most suitable method of approximating ovarian volume. Clinicians and researchers use a myriad of techniques ranging from semi-automated volumetric task functions offered by conventional ultrasound systems to manual calculations using linear measurements made in multiple cross-sectional images. In the present study, we employed the equation for a prolate spheroid, rather than the commonly used equation of a prolate ellipsoid, since this method was found to correlate better with volume measurements of polycystic ovaries made by 3D ultrasound [22].

Historically, the peripheral distribution of follicles has been considered a hallmark of polycystic ovaries [16]. The classic "string of pearls" appearance is embedded in the Medical Imaging literature and remains highly remarked upon in radiological reports confirming the presence of polycystic ovarian morphology. In the current study, determination of follicle pattern among observers was poor. Difficulty assigning follicle pattern may have related to confusion over the most appropriate ovarian cross-section in which to make the determinations since observers were analyzing digital recording rather than static images. Moreover, there may have been reluctance to assign follicle pattern in the presence of a dominant follicle or CL. We were unable to find any study reporting specific reliability coefficients when assigning follicle pattern using static or dynamic transvaginal ultrasonography [17]. While the current ultrasound criteria for polycystic ovaries exclude an assessment of follicle pattern, the appropriateness of its omission as a diagnostic criterion is questionable. Recently, a surrogate and more objective measure of follicle pattern, called the stromal-total area ratio, was shown to have 100% specificity and 100% sensitivity in diagnosing polycystic ovaries [36]. This group also recently reported good reliability among observers when making calculations of the stromal-total area ratio [37]. We suspect that wider adoption of this criterion may occur in light of favorable reports pertaining to its ease of use in clinical practice [37].

Agreement in the identification of CL was good among observers. Disagreement among observers was generally noted only when a CL appeared as a cystic structure rather than a hyperechoic structure with a small to negligible fluid-filled cavity [38]. In these instances, there was a tendency to mistake a CL for a dominant follicle (i.e., accounting for outlier measurements for the largest follicle diameter endpoint). Identifying the presence of CL is a highly important finding given its implications for infertility and risk of endometrial hyperplasia. However, it has been our experience that very few ultrasound reports comment on the presence or absence of a CL leading one to suspect that identification of ovulatory structures is not part of routine radiological assessments for many practices. While CL are generally present during the luteal phase, it is important to note that CL (albeit non-functional) can be visualized ultrasonographically during the early follicular phase [38]. This coincides with the recommended time for the ultrasonographic evaluation of PCOS [17]. Given growing recognition that some women with PCOS demonstrate regular menses, it is important to corroborate any evidence of ovulation to ascertain potentially lower health risks in this discrete subset of patients [39].

While it is tempting to conclude that levels of agreement reported in this study were due to differences in experience (i.e., three of four observers were trainees), it is important to recognize that all observers were deemed experienced gynecological ultrasonography. In the case of the radiologists, both were senior Radiology residents that had fulfilled the ultrasonographic requirements for their training programs and were scheduled to enter general practice in less than a year. In the case of the reproductive endocrinologists, one was a gynecologist with more than twenty years of ultrasonography experience while the other was a fellow who at the time of the study had more than 18 months of intensive training in ovarian ultrasonography. Better agreement among reproductive endocrinologists could be due to the fact that both were working together at the same institution, in an area of study where there was greater likelihood of encountering polycystic ovarian morphology. Nevertheless, it should be noted that overall levels of agreement were highest among Observers 1 and 2 – a reproductive endocrinologist and a radiologist – suggesting that discipline alone cannot fully explain the disparity among groups. While Observer 3 may have lessened agreement among radiologists by undercounting follicles and overestimating follicle size, this observer's conservative approach surely represents a subset of Medical Imaging specialists that would interpret ultrasonographic images of polycystic ovaries in a similar fashion. Ultimately, this set of observers is representative of a real-life clinical setting.

In summary, inter-observer agreement for identifying and quantifying individual ultrasonographic features of polycystic ovaries was moderate to poor. Agreement was best for the identification of a CL followed by determination of ovarian volume, largest follicle diameter, follicle distribution pattern and lastly, total follicle count. While we recognize that not all of these features are used to diagnose polycystic ovaries, we believe each of these features should be evaluated at the time of ovarian ultrasonography since each relates to an important aspect of ovarian physiology. If ultrasonographic evidence of polycystic ovaries is to be used as an objective measure in the diagnosis of PCOS, then decreasing variability in the ultrasound diagnosis is crucial. Standardized training modules for the uniform acquisition and interpretation of ultrasonographic images may be a necessary first step toward improving reliability in identifying polycystic ovarian morphology.

## Competing interests

The authors declare that they have no competing interests.

## Authors' contributions

MEL conceived, designed and coordinated the study, performed the ultrasound scans, conducted the statistical analyses and drafted the final manuscript. DRC clinically evaluated the study volunteers for PCOS. DRC, AKP, AD and MEL performed the post-hoc sonographic evaluations. RAP participated in the conception and design of the study and provided resources and equipment to complete the study. All authors read and approved the final manuscript.
